# Angiopoietins as Predictor Indexes in COVID-19 Patients in Delta and Omicron Waves

**DOI:** 10.3390/cimb46050245

**Published:** 2024-04-26

**Authors:** Panagiota Tsiatsiou, Kyriakos Kouirouxis, Vasiliki Tsaireli, Antonia Lanta, Angeliki Kassomenaki, Maria Papaioannou, Efthymia Protonotariou, Lemonia Skoura

**Affiliations:** 1 Department of Microbiology, AHEPA University Hospital, School of Medicine, Aristotle University of Thessaloniki, 54124 Thessaloniki, Greece; kouirouxis@hotmail.gr (K.K.); vtsaireli@yahoo.gr (V.T.); tonia1985@msn.com (A.L.); angelikikasso@gmail.com (A.K.); protonotariou@gmail.com (E.P.); mollyskoura@gmail.com (L.S.); 2 Division of Hematology, First Department of Internal Medicine, AHEPA General Hospital, Aristotle University of Thessaloniki, 54636 Thessaloniki, Greece; marypap@gmail.com

**Keywords:** Angiopoietin-1 (Ang-1), Angiopoietin-2 (Ang-2), COVID-19, endothelial dysfunction

## Abstract

This study aimed to explore the correlation between Angiopoietin-1 (Ang-1) and Angiopoietin-2 (Ang-2) concentrations and the Angiopoietin-2/Angiopoietin-1 ratio (Ang-2/Ang-1) with clinical outcomes, potentially serving as disease severity and survival biomarkers. A study at AHEPA University Hospital involved 90 Coronavirus Disease 2019 (COVID-19) adult patients, 30 hospitalized intensive care units (ICU), 30 inward units (non-ICU), and 30 asymptomatic non-hospitalized individuals as controls. Estimated endothelial dysfunction markers related to angiogenesis were measured. There was a statistically significant difference only between outpatient and hospitalized patients (non-ICU–ICU groups) for the Ang-1 and Ang-2 indices. The Ang-2/Ang-1 ratio has differed significantly among the individual patient groups. An ROC analysis was conducted to find an optimal threshold for distinguishing between (outpatients–non-ICU) and (non-ICU–ICU) groups. It was based on Youden’s index of 0.1122 and 0.3825, respectively. The Ang-1, Ang-2 levels, and Ang-2/Ang-1 ratio were analyzed as severity indicators in COVID-19 patients. The Ang-2/Ang-1 ratio demonstrated better prognostic and diagnostic utility than individual biomarker levels. Monitoring the Ang-2/Ang-1 ratio can identify COVID-19 patients at risk and assist clinicians in tailoring treatment strategies to improve outcomes.

## 1. Introduction

Originating in Wuhan, China, in early December 2019, coronavirus disease 19 (COVID-19) has spread rapidly, with cases confirmed in every country. Coronavirus disease 2019 (COVID-19), caused by the SARS-CoV-2 infection, belongs to the subfamily Coronavirinae in the family Coronaviridae [1]. It is still a persistent threat to global public health, resulting in widespread morbidity and mortality and highly intensive care unit use. SARS-CoV-2 targets both the respiratory and extrapulmonary systems. The clinical symptoms varied in severity from mild to severe cases. The disease is characterized by pneumonia, fatigue, fever of 38 °C or higher, cough, dyspnea, headache, diarrhea, vomiting, abdominal pains, fatigue, and muscle pains, as well as changes in taste and smell [2]. 

As for the virus, SARS-CoV-2 has a single-stranded RNA genome that is encapsulated has a positive sense. As a result, it is exposed to not only recurring point mutations due to selective pressure but also a progressive accumulation of mutations over an extended period. When it infects a host cell, it utilizes the host’s protein synthesis machinery to generate viral proteins necessary for reproduction.

During this pandemic, novel variants of SARS-CoV-2 have emerged. The World Health Organization (WHO) classified some variants as variants of interest (VOIs) and variants of concern (VOCs). Variants of interest (VOI) are those that exhibit features that warrant continuous monitoring and additional analysis, while variants of concern (VOC) have been demonstrated to be more virulent, transmissible, pathogenic, and may potentially evade treatment options and/or immune responses due to vaccination or previous infection [3].

The Delta variant prevailed during the 4th wave of the epidemic in Greece, from mid-summer to the end of 2021, followed by the Omicron variant, and the 5th wave was the period during which the Omicron variant was dominant (approximately January to May of 2022). Despite its milder severity, the Omicron variant is more contagious than the Delta variant [4,5]. During this period, immunity was based on protection from prior vaccination and infection [6].

Through the ACE2 receptor, SARS-CoV-2 virus targets the pulmonary system primarily. The ACE2 receptor exhibits significant expression in diverse organs and tissues, facilitating the virus’s ability to disseminate to different organs and infect cells that express ACE2 at localized locations. COVID-19 is linked to extensive injury to endothelial cells and epithelial cells in lung tissue, leading to increased permeability of capillaries, infiltration of inflammatory cells into tissues surrounding blood vessels, retention of fluid in the extracellular spaces, and the development of acute respiratory distress syndrome (ARDS). In acute lung injury, ARDS is the most severe manifestation, and it is lethality-related to COVID-19. Also, in severe cases, it can lead to acute kidney injury, heart injury, and liver failure. COVID-19 can cause acute lung injury, myocardial infarction, liver injury, kidney injury, and other injuries. Endothelium dysfunction is evidence of COVID-19 syndrome [7,8].

COVID-19-associated endotheliopathy was promptly acknowledged at the onset of the disease. Postmortem investigations unveiled the direct invasion of endothelial cells by SARS-CoV-2 or indirectly cause injury through a cytokine storm. Severe cases of COVID-19 are characterized by hyper-inflammatory and thrombotic episodes, cell death, and endothelial dysfunction, suggesting that one of the major targets of this disease is the endothelium, one of the body’s largest organs.

Endothelial cells, a part of blood vessels, maintain micro and macrovascular health by identifying pathogens and secreting vasoactive molecules [9]. In addition, endothelial cells release pro-fibrinolytic agents that initiate fibrinolysis to degrade clots [10]. Recent understanding reveals endothelium’s role in regulating blood vessel tone, cell behavior, and innate immunity [11].

Macro- and microvascular thromboembolic or in situ thrombotic complications have been observed in COVID-19 patients in the vasculature of the lungs, spleen, brain, gut, and periphery. Pulmonary embolism (PE) and deep vein thrombosis constitute the most frequent thrombotic events. Additionally, in patients diagnosed with COVID-19, there was an increased occurrence of alveolar capillary microthrombi, nine times greater than that in patients with influenza. This finding aligns with the higher frequency of blood clot formation observed in COVID-19 cases than in other viral lung infections. Severe endothelial injury and intracellular viruses were also noted in patients with COVID-19 at sites where microthrombosis was observed, indicating that the inflammation and injury to the endothelial cells may directly contribute to the formation of blood clots. Moreover, thromboses have been observed both during the early stage of the disease and in the following weeks, suggesting that the tendency towards a prothrombotic condition may persist for several weeks or even post-hospitalization [12].

The endothelial Angiopoietin/Tie2 system is involved in regulating endothelial permeability. Endothelial cells primarily express and contain the tyrosine kinase receptor Tie2. The activity of Tie2 is modulated by its ligands, Ang-1 and Ang-2, which are vascular growth factors that contribute to the process of angiogenesis. They act in an agonist/antagonist manner and may directly or indirectly alter the structure and integrity of the vascular endothelium directly or indirectly [13,14].

Angiopoietin 1 (Ang-1) promotes barrier function by reorganizing endothelial cells. During inactivation, pericytes secrete (Ang-1), which acts as a Tie2 agonist and contributes to maintaining an effective endothelial barrier. Depending on the circumstances, Angiopoietin-2 (Ang-2) is either an antagonist or a partial agonist of the Tie2 receptor. Weibel–Palade bodies release (Ang-2) during pathological conditions, which antagonistically binds to the Tie2 receptor [15]. Thus, downstream Tie2 signaling is deactivated, cell–cell junctions deteriorate, and endothelial hyperpermeability and vascular leakage are induced. Therefore, endothelial Angiopoietin/Tie2 system alterations may contribute to organ injury during critical illness. Therefore, endothelial Angiopoietin/Tie2 system disruptions may contribute to organ damage during critical illness [13,16]. In response to endothelial dysfunction, endothelial cells initiate the formation of fibrin, as well as the adhesion and aggregation of platelets [10].

This study aimed to identify the appropriate prognostically significant biomarkers and provide the ability to stratify the risk of thrombosis in COVID-19 patients. Many studies have suggested that an increase in Ang-2 and a decrease in Ang-1 may be indicative of a pro-inflammatory state that is best described by the Ang-2 to Ang-1 ratio [12,17]. Considering these findings, our primary objective was to examine the relationship between decreased Ang-1 and increased Ang-2 concentrations, the Ang-2/Ang-1 ratio, and clinical outcomes [18,19]. They may appear valid as disease severity and survival biomarkers are prospective and promising therapeutic targets.

## 2. Materials and Methods

### 2.1. Study Design and Patient Characteristics

This single-center, cross-sectional, observational study included 90 COVID-19 adult patients (≥18 years) and was conducted at the AHEPA University Hospital, a 700-bed tertiary care hospital in Thessaloniki, Greece, from 20 July 2021 to 30 December 2022. During the pandemic, the AHEPA hospital served as one of the reference hospitals for COVID-19 patients in Northern Greece. The study included 60 patients who fulfilled the criteria for hospitalization (30 patients with COVID-19 hospitalized in intensive care units, and 30 patients with COVID-19 disease hospitalized in ward units) and 30 asymptomatic patients, non-hospitalized individuals with COVID-19 disease, who were used as controls for biomarker measurements.

The diagnosis of COVID-19 was based on real-time reverse transcription PCR (RT-PCR) of nasopharyngeal swab samples. The study was approved by the AHEPA Hospital Research Ethics Committee (129/19 March 2020, and the bioethics approval committee of Aristotle University. All the procedures were carried out in patients in compliance with the Declaration of Helsinki. Informed written consent was obtained from all patients prior to any study procedure. Patients younger than 18 years old, with a history of cancer, a diagnosis of hematological disease or thrombophilia, liver disease or renal failure, or a critical illness related to another diagnosis other than COVID-19 that affects the thrombotic status of the patients, were not included in the study.

### 2.2. Blood-Sampling Procedures

Venous blood was collected with the first scheduled morning draw within 72 h post-ward and ICU admission. Based on the test, blood samples were drawn into plastic vacutainer tubes containing 0.129 M (3.8%) trisodium citrate (catalog number 363095; Becton Dickinson, Dublin, Ireland) for plasma samples in a 9:1 blood-to-anticoagulant ratio. Test samples were centrifuged twice at 2500× *g* for 15 min at 18–25 °C to obtain poor platelet plasma. Venus blood samples were also collected in vacutainer tubes coated with silicon and micronized silica particles to accelerate clotting for procedures that require serum samples (catalog number 367837; Becton-Dickinson, Dublin, Ireland). Serum was collected, portioned into 0.5 mL aliquots, and stored at 80 °C until used. All samples were frozen until used. Freeze–thaw cycles were avoided.

### 2.3. Laboratory Procedures

The routine clotting assays, including prothrombin time (PT), activated partial thromboplastin time (aPTT), fibrinogen, and D-dimers, were performed in the ACL TOP 50 series (Instrumentation Laboratory, Bedford, MA, USA), with manufacturer reagents and controls per laboratory protocol.

Endothelial dysfunction and angiogenesis laboratory markers, Ang-1 and Ang-2 were measured in the serum by enzyme-linked immunosorbent assay (ELISA) according to the manufacturer’s instructions (R&D Systems Inc., Minneapolis, MN, USA). For Ang-1 (sensitivity: 10.30 pg/mL, range: 14,272–65,570 pg/mL, mean value: 37,122 pg/mL, cross-reactivity < 0.50%) and Ang-2 (sensitivity: 21.30 pg/m, range: 1065–8907 pg/mL, SD: 1341 pg/mL, cross-reactivity < 0.50%). A 50-fold dilution was suggested for Ang-1 and a 5-fold dilution for Ang-2 serum samples, respectively. All samples were assayed in duplicate. Biomarkers are presented as their standard reference ranges and ratio. Normal values for Ang-1 and Ang-2 were 14,272–65,570 pg/mL and 1065–8907 pg/mL, respectively. The analysis relies upon the utilization of patients’ biomarker levels, i.e., Ang-1 and Ang-2 patient levels, and the corresponding ratio (Ang-2/Ang-1 index).

### 2.4. Statistical Analysis

The data are presented as the median along with the interquartile range (IQR), explicitly ranging from the first quartile (Q1) to the third quartile (Q3), which is particularly suitable for skewed data. For the quantitative indices of the study, descriptive statistics were estimated for the entire sample and the individual groups of patients, that is, outpatient, non-ICU, and ICU. Additionally, the Kolmogorov–Smirnov test for normality was performed. Plasma levels of Ang-1, Ang-2, and the Ang-2/Ang-1 ratio, exhibiting non-normal distributions, are subject to comparison across the three patient groups using the Mann–Whitney U rank sum test—a non-parametric approach.

To identify the most effective threshold for distinguishing between the three groups of patients, a Receiver Operating Characteristic (ROC) curve analysis was performed using the Ang-2/Ang-1 index. The area under the curve (AUC) is calculated to assess the accuracy of this discrimination. Further, Youden’s index, which is a commonly used statistic that aims to find a threshold that maximizes the difference between sensitivity and (1-specificity), is utilized. It is calculated for each threshold by adding sensitivity and specificity and subtracting 1. The threshold corresponding to the highest Youden’s index was a potential cut-off point.

The statistical analysis of the data was conducted using IBM SPSS Statistics, version 29.

## 3. Results

Patient characteristics, such as age (mean, 43 years; range, 27–75 years), sex (15 males and 15 females for each group), and demographic characteristics (Caucasians), were not significantly different between the groups (Table 1) Also, in Table 1, the vaccination of the patients is shown. Patient comorbidities are reported in Table 2. Further, mean-SD for standard laboratory tests in a total number of patients, outpatients, non-ICU, and ICU are shown in Table 3. Specifically, 30 patients with COVID-19 were hospitalized in intensive care units (ICU), 30 patients with COVID-19 outside of intensive care units in the hospital wards (non-ICU), and 30 asymptomatic non-hospitalized patients with COVID-19 (outpatients). 

### 3.1. Results for Ang-1

**Figure 1:**
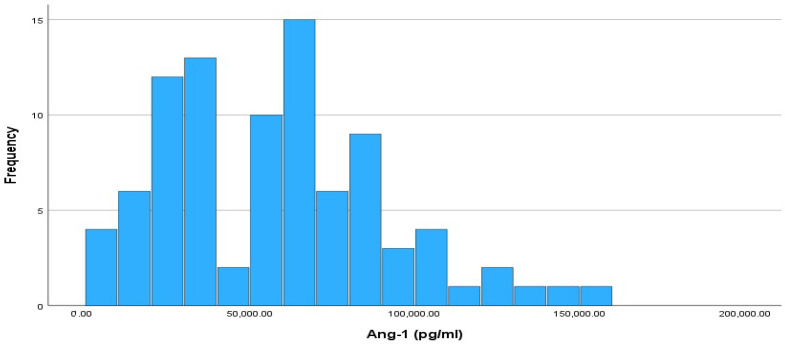
Histogram of absolute frequencies for Ang-1 level. Sample size is n = 90.

Figure 1 shows a histogram of the absolute frequencies of Ang-1. The Kolmogorov–Smirnov test indicated that the Ang-1 index did not follow a normal distribution (*p*-value = 0.012 < 0.05). The median value of Ang-1 was 54,525 pg/mL, while the interquartile range was 49,422.50 pg/mL, with IQR limits of 30,340 pg/mL (Q1) and 79,762.50 pg/mL (Q3).

**Figure 2:**
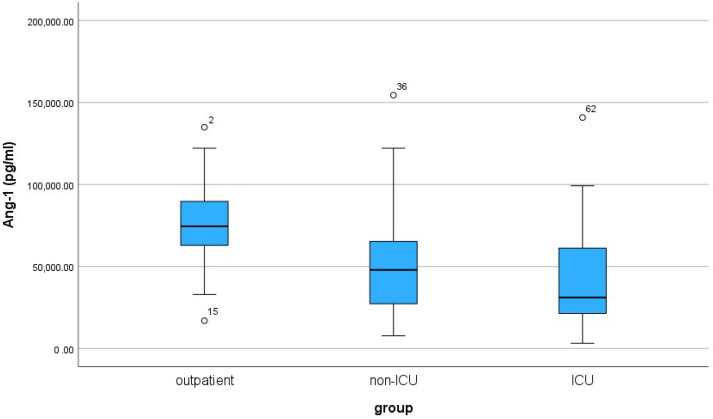
Box plot of Ang-1 for the three groups of patients: outpatient, non-ICU and ICU. Sample size is n = 30 for each patient group. The “o” represents outliers.

For the three patient groups, the median and IQR values are shown in Table 4. Ang-1 levels varied across the three patient groups, with outpatients showing the highest median (74,450 pg/mL) and the least variability (IQR 28,812.5 pg/mL). Non-ICU patients had a lower median (47,890 pg/mL) and a wider IQR (40,177.5 pg/mL), while ICU patients exhibited the lowest median (31,070 pg/mL) and the widest IQR (41,852.5 pg/mL). The boxplot of Ang-1 visually depicts the distribution and comparative analysis of Ang-1 levels across outpatients, non-ICU, and ICU groups (Figure 2).

According to the non-parametric Mann–Whitney U rank test, significant variations in Ang-1 levels emerged between outpatient and non-ICU patients (*p*-value < 0.001), where outpatients demonstrated a significantly higher median value than non-ICU patients. There was also a statistically significant difference between the outpatient and ICU patient (*p*-value < 0.001) groups. In contrast, the test showed no significant difference between the non-ICU and the ICU groups (*p*-value = 0.243) regarding the Ang-1 index.

The significant differences in Ang-1 levels observed between outpatients and both non-ICU and ICU groups suggest that Ang-1 possesses a discriminatory ability to distinguish outpatient conditions from inpatient settings. However, the lack of a significant difference between the non-ICU and ICU groups indicated a potential limitation in Ang-1’s ability to discriminate between these two inpatient categories.

### 3.2. Results for Ang-2

**Figure 3:**
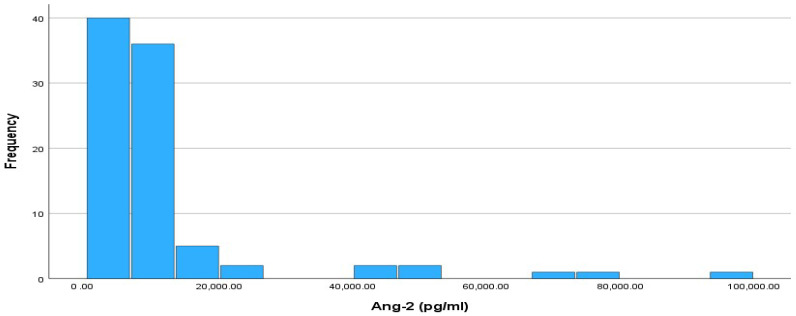
Histogram of absolute frequencies for Ang-2. Sample size is n = 90.

Figure 3 shows a histogram of the absolute frequencies for Ang-2. In addition, according to the normality test for the Ang-2 index, it did not follow a normal distribution according to the Kolmogorov–Smirnov test (*p*-value < 0.001). The median value of Ang-2 is 7419 pg/mL, while the interquartile range (IQR) is 5089.75 pg/mL, with IQR limits of 5110.25 pg/mL (Q1) and 10,200 pg/mL (Q3).

**Figure 4:**
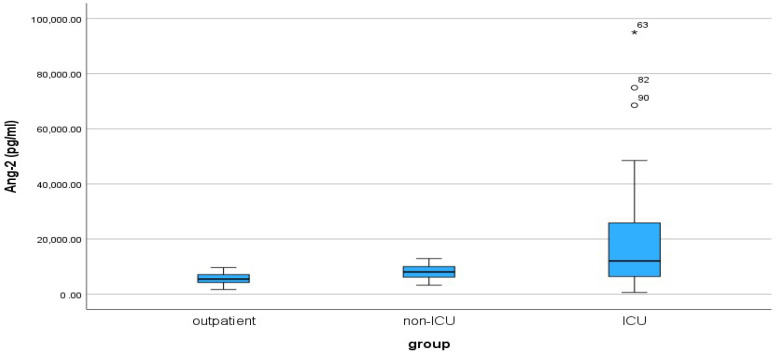
Box plot of Angiopoietin-2 for the three groups of patients: outpatient, non-ICU and ICU (Intensive Care Unit, ICU). Sample size is n = 30 for each patient group. The “o” represents outliers, while “*” represents extreme outliers.

The median and the interquartile range (IQR) values for the three groups of patients are shown in Table 5. The median Ang-2 levels exhibited a gradient among the patient groups, with outpatients showing the lowest median (5475.00 pg/mL), followed by non-ICU patients (8057.50 pg/mL), and ICU patients with the highest median (12,038.50 pg/mL). The considerable interquartile ranges within each group underscored the notable variability in Ang-2 levels across these distinct patient populations. A boxplot of Ang-2 across the three groups of patients is shown in Figure 4.

According to the non-parametric Mann–Whitney-U test, there was a statistically significant difference between the outpatient-non-ICU and outpatient-ICU groups (*p*-value < 0.001). In contrast, the non-ICU and ICU groups did not differ significantly regarding the Ang-2 index (*p*-value = 0.060).

Ang-2 displays a notable discriminative ability, as evidenced by its significant capacity to differentiate between outpatients and inpatient groups. However, the lack of a significant difference between non-ICU and ICU patients suggests a potential limitation in its discriminatory power within the inpatient setting, highlighting the nuances in its ability to distinguish between specific categories of patients.

### 3.3. Results for Ang-2/Ang-1 Ratio

**Figure 5:**
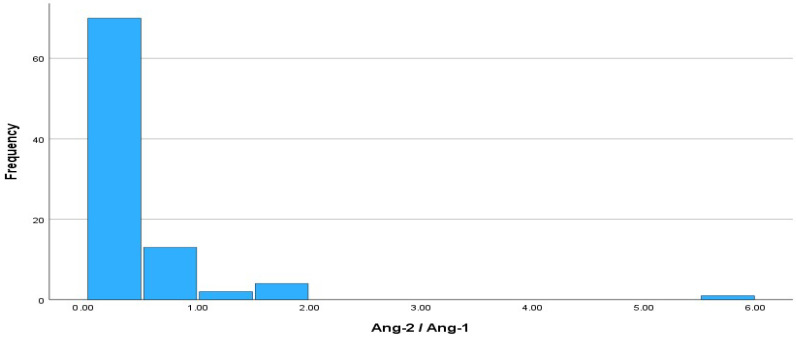
Histogram of absolute frequencies for Ang-2/Ang-1 ratio. Sample size is n = 90.

A histogram depicting the absolute frequencies of the Ang-2/Ang-1 ratio is presented in Figure 5. Regarding the ratio, the Ang-2/Ang-1 variable did not follow a normal distribution (Kolmogorov–Smirnov test, *p*-value ≤ 0.001). The median value of the Ang-2/Ang-1 ratio was 0.1465, and the interquartile range (IQR) was 0.35, with IQR limits of 0.0776 (Q1) and 0.4265 (Q3).

**Figure 6:**
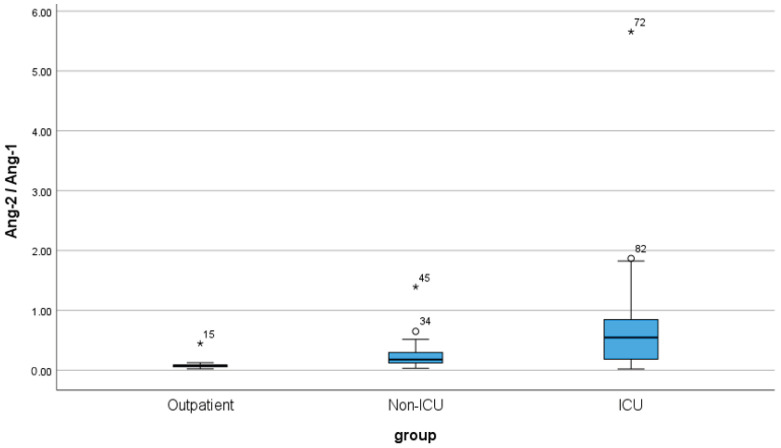
Box plot of Ang-2/Ang-1 for the three groups of patients: outpatient, non-ICU, and ICU (Intensive Care Unit, ICU). Sample size is n = 30 for each patient group. The “o” represents outliers, while “*” represents extreme outliers.

Table 6 presents the median and IQR values of the three groups of patients. The Ang-1/Ang-2 ratio demonstrates an upward trend, progressing from outpatients (median: 0.0767, IQR: 0.03) to non-ICU patients (median: 0.1790, IQR: 0.18) and peaking in ICU patients (median: 0.5455, IQR: 0.68). These findings imply a potential association between the ratio and the severity of the medical condition, with elevated values observed in more critically ill patients. The boxplot depicting the Ang-2/Ang-1 ratio variation among the three patient groups vividly illustrates the observed trends (Figure 6).

According to the non-parametric Mann–Whitney U test, there was a statistically significant difference among the individual patient groups for the Ang-2/Ang-1 ratio: outpatient–ICU (*p*-value < 0.001), outpatient–non-ICU (*p*-value < 0.001), and non-ICU–ICU (*p*-value = 0.002).

The Ang-1/Ang-2 ratio demonstrated a progressive increase in median values across the outpatient, non-ICU, and ICU groups, indicating a potential association with disease severity. This suggests that the ratio may serve as a valuable marker for distinguishing patients based on their clinical status, with higher values suggesting more critical conditions. Furthermore, the Mann–Whitney U test revealed statistically significant differences in the Ang-2/Ang-1 ratio among all pairs of patient groups. This indicated distinct patterns in the Ang-2/Ang-1 ratio across different clinical settings, highlighting its potential utility as a discriminatory marker for patient stratification.

### 3.4. Results of ROC Analysis and Ang-2/Ang-1 Ratio

**Figure 7:**
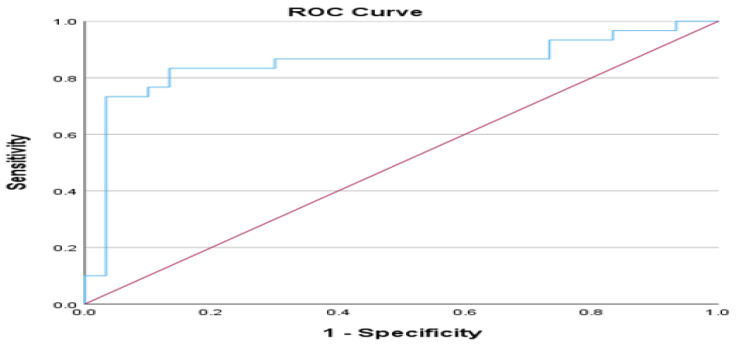
Receiver Operating Characteristic (ROC) curve for Ang-2/Ang-1 ratio for outpatient and non-ICU patients, area under the ROC curve (AUC) 0.849, 95% Confidence Interval from 0.738 to 0.959, *p*-value < 0.001. Blue line represents sensitivity vs. 1 − specificity (1 minus specificity), while the red line represents the diagonal, i.e., the line of no discrimination or the reference line. Sample size is n = 30 for each patient group.

Exploring the categories of outpatient and non-ICU patients, we conducted an ROC analysis utilizing the Ang-2/Ang-1 ratio to ascertain the optimal threshold for distinguishing between these groups. The area under the curve (AUC) attained a value of 0.849 (*p*-value < 0.001), indicating that the model is effective for classification tasks. The asymptotic 95% Confidence Interval ranged from 0.738 to 0.959, further underscoring the robustness of these findings. Based on the extracted results, the optimal cut-off point based on Youden’s index was 0.1122 (sensitivity = 0.833, specificity = 0.867). Figure 7 illustrates the ROC analysis for the Ang-2/Ang-1 ratio in the outpatient and non-ICU patients.

**Figure 8:**
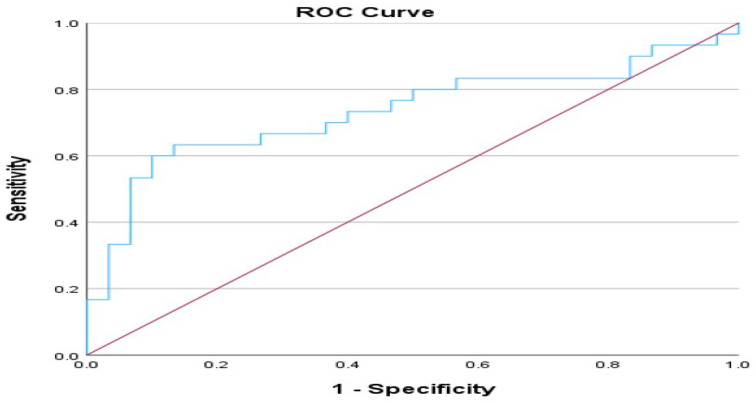
Receiver Operating Characteristic (ROC) curve for Ang-2/Ang-1 ratio for non-ICU and ICU patients, area under the ROC curve (AUC) 0.734, 95% Confidence Interval from 0.602 to 0.867, *p*-value = 0.002. Blue line represents sensitivity vs. 1 − specificity (1 minus specificity), while the red line represents the diagonal, i.e., the line of no discrimination or the reference line. Sample size is n = 30 for each patient group.

Furthermore, we conducted an ROC analysis based on the Ang-2/Ang-1 ratio comparing non-ICU and ICU patients. The Area Under the Curve (AUC) attained a value of 0.734 (*p*-value = 0.002), indicating that the model is acceptably effective in classification tasks. The asymptotic 95% confidence interval ranged from 0.602 to 0.867. Youden’s index was utilized to extract a potential cut-off point of 0.3825 (sensitivity = 0.633, 1 − specificity = 0.133). An ROC analysis of the Ang-2/Ang-1 ratio in non-ICU–ICU patients is shown in Figure 8.

## 4. Discussion

Different conditions, such as diabetes mellitus, atherosclerosis, hypertension, endothelial dysfunction, and impaired angiogenesis, contribute to microvascular dysfunction [11,19]. It has been demonstrated that markers of endothelial dysfunction and angiogenesis are predictors of disease severity in heart failure, renal failure, and after cardiac surgery [20,21]. These biomarkers circulate in the blood and can cause and affect microvascular dysfunction.

Therefore, it is crucial to identify valuable biomarkers to enhance early diagnosis and risk stratification. In our study, we analyzed the Angiopoietin-1 and Angiopoietin-2 indexes and Angiopoiten-2/Angiopoiten-1 ratio as indicators that will serve as a screening tool for severity in COVID-19 patients in delta and omicron waves. Regarding the Angiopoietin-1 and Angiopoietin-2 indices, we found significant differences between the outpatient and hospitalized patients (non-ICU and ICU groups). In contrast, the non-ICU–ICU groups did not differ significantly. In addition, for the Ang-2/Ang-1 index, there was a significant difference among the individual patient groups: outpatient–non-ICU, outpatient–ICU, and ICU–non-ICU.

Furthermore, an ROC analysis using the Ang-2/Ang-1 index was conducted to ascertain the optimal threshold for distinguishing between groups. Between the outpatient and non-ICU patient categories, the AUC was 0.849, indicating the model’s effectiveness. The 95% confidence interval ranged from 0.738 to 0.959, indicating robustness. Youden’s index suggested an optimal cut-off point of 0.1122. Furthermore, the ROC analysis showed an acceptable model for classification tasks between the non-ICU and ICU groups, with an AUC of 0.734 and a potential cut-off point of 0.3825. However, the Ang-2/Ang-1 ratio in our research was regarded as an essential biomarker for rapid risk stratification and prognosis. The ratio of Ang-2 to Ang-1 demonstrated excellent prognostic and diagnostic utility, surpassing the levels of Ang-1 and Ang-2 indices. Moreover, this ratio demonstrated notable diagnostic and prognostic efficacy for predicting severity.

In addition to supporting the value of decreasing levels of Angiopoietin-1 as an indicator for hospitalization, particularly when comparing patients requiring hospital care to those who can be managed as outpatients, our research reinforces prior findings. Researchers in two separate studies, Abou-Arab et al. and Vassilliou et al., have provided additional insights that there is no difference in Ang-1 levels between patients with severe and critical COVID-19 patients and survivors and non-survivors. In our study, there was no difference between hospitalized patients (non-ICU–ICU patients) [22,23]. We also assessed the endothelial damage resulting from increased levels of Ang-2 levels. This discovery reinforces the theory that COVID-19 is a vascular condition characterized by injury to the endothelium, angiogenesis, and thrombosis.

According to Six et al. and Alay et al., circulating levels of Angiopoietin-2 as a marker of endothelial injury were higher in hospitalized patients with COVID-19 than in healthy controls [6,24]. Also, the study by Smadja et al. indicated that Angiopoietin-2 is a relevant predictive factor for ICU direct admission in COVID-19 patients [25]. Henry et al. as well found that increased Ang-2 expression is related to endothelial activation and an increased risk of ICU admission in patients admitted with COVID-19 [26,27]. In addition, three other studies have reported the significant role of Ang-2. In the first study, Abou-Arab et al. found that Ang-2 levels were significantly higher in the critical group [22]. In the second study, Vill et al. showed a strong correlation between the Angiopoietin-2 course and hospital mortality and a non-resolving pulmonary condition associated with COVID-19 [28]. In the third study, Kümperset al. highlighted the independent prognostic impact of circulating Ang-2 levels in critical illness [29].

In another study by Vassilliou et al., patients who were directly admitted to the ICU rather than the ward had elevated levels of Ang-2 [23]. In our research, Angiopoietin-2 was a valuable indicator for the hospitalization of patients in comparison to the outpatient and hospitalized groups, not only in critical care patients. The discrepancies between our observations and the aforementioned studies and others may be due to the emergence of new virus variants, and the availability of new therapies and vaccines may lead to different outcomes.

Ong et al.’s research reported that the Ang-2/Ang-1 ratio may be a prognostic biomarker of endothelial activation in patients with acute lung injury and may also be helpful for risk stratification in these patients [30]. Also, in the survey by Gouzi et al., the Ang-2/Ang-1 ratio is a potential marker of vascular impairment in chronic obstructive pulmonary disease (COPD) patients [31].

Moreover, according to the findings of Fang et al., Angiopoietin-2/Angiopoietin-1 and ratios could have a predictive value for early sepsis in an emergency department [17]. In a study by Lázaro et al., the Ang-2/Ang-1 ratio was shown to be a predictor of severity in critically ill patients [19]. According to our research, the Ang-2/Ang-1 ratio is a potent severity indicator that is considerably higher in the critical group of COVID-19 patients, consistent with Abou-Arab et al. research findings [22].

Monitoring the ratio of Ang-2 to Ang-1 in COVID-19 patients could aid in identifying those at a higher risk for critical outcomes and engaging in treatment decisions. Modifications in the levels of Ang-1 and Ang-2 and the Ang-2/Ang-1 ratio may indicate treatment efficacy and serve as plausible therapeutic objectives for multi-targeted therapies.

Our study agrees with Seol et al.’s results for sepsis [32]. Ang-1 and Ang-2 concentrations correlate with disease severity and survival, as their predictive values indicate. The potential ability of Ang-1 and Ang-2 concentrations to differentiate is clinically significant and should be further explored before implementation in routine practice.

The limitations of our study include the fact that (1) the patient selection process was conducted randomly without considering specific variables that may have influenced the indices, such as diabetes mellitus and chronic obstructive pulmonary disease, etc. (2) Additionally, cases pertaining to the α wave were not incorporated into the study. (3) As an agonist or antagonist of the Tie-2 receptor, Ang-2 has also been shown to be context-dependent, and its role may depend on other growth factors, such as VEGF-A. We did not measure VEGF-A and Tie-2 receptors in our cohort, so we were unable to corroborate their role in relation to Angiopoietins. (4) Smoking status has not been reported. (5) Finally, the small cohort size conducted in a singular center prevents us from extracting broad conclusions from our data. In addition, there was a lack of information including anthropometric data for the patients. To confirm and extend these findings, we proposed that the Ang-2/Ang-1 ratio be validated as a prognostic factor in a larger cohort of patients. Future research should investigate the relationship between Ang-2/Ang-1 and other endothelial permeability markers to improve the prediction of severity and stratification, especially in diseases with endothelial dysfunction.

## 5. Conclusions

We have identified circulating vascular markers that can be used to monitor disease severity in COVID-19 patients in the delta and omicron waves. Monitoring the Ang-2/Ang-1 ratio could aid in identifying COVID-19 patients at a higher risk of critical outcomes. Moreover, these markers will assist clinicians in tailoring treatment strategies for improved outcomes. All hospitalized COVID-19 patients exhibited an increase in angiogenesis markers, which might suggest the presence of hypoxemia and inflammation. Endothelial cell integrity provides an antithrombotic environment, and the findings of pulmonary angiopathy in patients with severe COVID-19 highlight the involvement of endotheliopathy in pulmonary perfusion defects, coagulopathy, and thrombosis.

As vaccine-resistant variants are likely to emerge, future studies must use biomarkers correlating with severity. In addition, the findings of our investigation support the hypothesis that COVID-19 induces microvascular dysfunction by activating the endothelium. Moreover, it is a crucial factor not only in COVID-19 but also in various other diseases such as hypertension, cardiovascular disease, diabetes, obesity, and endothelial dysfunction. This dysfunction plays a substantial role in disease progression, particularly in vascular issues and thrombosis. Therefore, a better understanding of the role of the endothelium can provide new possibilities for the exploration and innovative treatments for the management of thromboembolism.

## Figures and Tables

**Table 1:** Demographic characteristics and vaccination.

Group of Patients (n = 90 Patients)	Asymptomatic (n = 30 Patients)		Non-ICU (n = 30 Patients)		ICU (n = 30 Patients)	
Age	27–75 years (mean: 43 years)					
sex	15 males and 15 females for each group					
demographic characteristics	(Caucasians)					
	2021	2022	2021	2022	2021	2022
History of infection	first	14 first 1 second	first	first	first	first
Unvaccinated	15 (100%)	5 (33.3%)	8 (53.2%)	4 (26.6%)	12 (79.9%)	8 (53.2%)
Vaccination Χ1	-	1 (6.6%)	5 (33.3%)	1 (6.6%)	1 (6.6%)	1 (6.6%)
Vaccination X2	-	1 (6.6%)	2 (13.2%)	-	2 (13.2%)	1 (6.6%)
VaccinationX3	-	7 (46.6%)	-	10 (66.6%)	-	5 (33.3%)

**Table 2:** Patients-Comorbidities.

	Aymptomatic (30 Patients)	Non-ICU (30 Patients)	ICU (30 Patients)
CVD	-	11 (73.2%)	10 (66.6%)
COPD	2 (13.2%)	1(6.6%)	8 (53.2%)
Hematologic diseases	-	2 (13.2%)	1(6.6%)
Ca	-	-	2 (13.2%)
DM	-	-	3 (19.9%)
neurological disorders	-	3 (19.9%)	1 (6.6%)
autoimmune diseases	-	1 (6.6%)	1 (6.6%)
Death	-	6 (4 CVD/1 neurologial disorders/1 hematological disease	19 (6 CVD/9 COPD/1 neurologial disorders/ 1 hematological disease/1 DM/1 Ca
ARDS/Death	-	4/6	17/19

CVD: Cardiovascular diseases (Hypertension-heart disease), COPD: Chronic obstructive pulmonary disease, Ca: Cancer, DM: Diabetes mellitus, ARDS: Acute respiratory distress syndrome.

**Table 3:** Mean–SD for standard laboratory tests in total number of patients, outpatients, non-ICU, ICU.

	Total Number of Patients (n = 90)		Outpatients (n = 30)		Non-ICU (n = 30)		ICU (n = 30)		
	Mean	SD	Mean	SD	Mean	SD	Mean	Mean	Units
HB	12.72	9.17	13.5	2.0	11.9	1.8	12.7	12.7	g/dL
HCT	35.79	7.33	40.7	6.0	36.2	5.2	30.2	30.2	%
WBC	11.23	9.19	6.4	2.7	10.2	6.4	17.2	17.2	K/μL
Neutrophils absolute count	8.95	8.72	4.1	2.3	8.5	5.9	14.2	14.2	K/μL
Neutrophils %	70.94	19.39	60.7	15.5	78.1	10.8	74.5	74.5	%
Lymphocytes absolute count	1.66	2.11	1.5	0.8	1.2	0.7	2.2	2.2	K/μL
Lymphocytes %	19.59	243.06	25.5	10.3	18.1	18.5	14.8	14.8	%
PLT	16.83	106.83	222.3	62.7	243.9	132.0	263.5	263.5	K/μL
PT	13.32	2.41	12.3	1.5	12.7	2.2	14.8	14.8	s
INR	1.13	0.21	1.0	0.1	1.07	0.2	1.2	1.2	
aPTT	33.51	8.71	32.3	11.0	33.6	8.4	35.8	35.8	s
Fibrinogen	413.28	148.65	414.5	161.1	394.6	140.2	429.1	429.1	mg/dL
D-dimer	807.37	953.26	307.0	341.1	630.5	588.2	1464.2	1464.2	ng/mL
FVII	112.47	76.18	115.9	91.3	136.6	136.6	85.5	85.5	%
FVIII	197.41	84.03	125.6	70.5	204.8	204.8	256.7	256.7	%
vWAg	252.98	111.89	183.2	60.4	229.6	229.6	349.3	349.3	IU/dL
vWRecof	253.4	82.43	207.9	59.9	234.7	234.7	322.0	322.0	IU/dL

**Table 4:** Median, 1st quartile (Q1), 3rd quartile (Q3) and IQR (interquartile range IQR = Q3 − Q1) in pg/mL for Ang-1 for the three groups of patients. Sample size is n = 30 for each patient group.

Groups	Median	(Q1, Q3)	IQR = Q3 − Q1
outpatient	74,450	(62,837.50, 91,650)	28,812.50
non-ICU	47,890	(26,410, 66,587.50)	40,177.50
ICU	31,070	(21,360, 63,212.50)	41,852.50

**Table 5:** Median, 1st quartile (Q1), 3rd quartile (Q3) and IQR (interquartile range IQR = Q3 − Q1) in pg/mL for Ang-2 for the three groups of patients. Sample size is n = 30 for each patient group.

Groups	Median	(Q1, Q3)	IQR = Q3 − Q1
outpatient	5475.00	(4194.00, 7111.25)	2917.25
non-ICU	8057.50	(6045.00, 10,051.25)	4006.25
ICU	12,038.50	(6203.75, 30,193.75)	23,990.00

**Table 6:** Median, 1st quartile (Q1), 3rd quartile (Q3) and IQR (interquartile range IQR = Q3 − Q1) for Ang-2/Ang-1 ratio for the three groups of patients. Sample size is n = 30 for each patient group.

Groups	Median	(Q1, Q3)	IQR = Q3 − Q1
outpatient	0.0767	(0.0571, 0.0864)	0.0293
non-ICU	0.1790	(0.1217, 0.2999)	0.1782
ICU	0.5455	(0.1835, 0.8591)	0.6756

## Data Availability

The data presented in this study are available on request from the corresponding author. The data are not publicly available due to intellectual property concerns.

## Abbreviations

Angiopoietin-1 (Ang-1); Angiopoietin-2 (Ang-2); Angiopoietin-2/Angiopoietin-1 ratio (Ang-2/Ang-1); Coronavirus Disease 2019 (COVID-19); Intense Care Unit (ICU); Receiver operating characteristic (ROC) Analysis; Acute respiratory distress syndrome (ARDS); World Health Organization (WHO); variants of interest (VOIs); variants of concern (VOCs); Pulmonary embolism (PE); real-time reverse transcription PCR (RT-PCR); prothrombin time (PT); activated partial thromboplastin time (aPTT); enzyme-linked immunosorbent assay (ELISA); interquartile range (IQR); area under the curve (AUC); chronic obstructive pulmonary disease (COPD); first quartile (Q1); third quartile (Q3).

## References

[1] Wu F., Zhao S., Yu B., Chen Y.-M., Wang W., Song Z.-G., Hu Y., Tao Z.-W., Tian J.-H., Pei Y.-Y. (2020). A new coronavirus associated with human respiratory disease in China. Nature.

[2] Mohan B., Vinod N. (2020). COVID-19: An Insight into SARS-CoV2 Pandemic Originated at Wuhan City in Hubei Province of China. J. Infect. Dis. Epidemiol..

[3] Akkız H. (2022). The Biological Functions and Clinical Significance of SARS-CoV-2 Variants of Corcern. Front. Med..

[4] Vitiello A., Ferrara F., Auti A.M., Di Domenico M., Boccellino M. (2022). Advances in the Omicron variant development. J. Intern. Med..

[5] Antonelli M., Pujol J.C., Spector T.D., Ourselin S., Steves C.J. (2022). Risk of long COVID associated with delta versus omicron variants of SARS-CoV-2. Lancet.

[6] Liosi S., Tsiambas E., Maipas S., Papageorgiou E., Lazaris A., Kavantzas N. (2023). Mathematical modeling for Delta and Omicron variant of SARS-CoV-2 transmission dynamics in Greece. Infect. Dis. Model..

[7] Mokhtri T., Hassani F., Ghaffari N., Ebrahimi B., Yarahmadi A., Hassanzadeh G. (2020). COVID-19 and multiorgan failure: A narrativereview on potential mechanisms. J. Mol. Histol..

[8] Xu S.-W., Ilyas I., Weng J.-P. (2022). Endothelial dysfunction in COVID-19: An overview of evidence, biomarkers, mechanisms and potential therapies. Acta Pharmacol. Sin..

[9] Six I., Guillaume N., Jacob V., Mentaverri R., Kamel S., Boullier A., Slama M. (2022). The Endothelium and COVID-19: An Increasingly Clear Link Brief Title: Endotheliopathy in COVID-19. Int. J. Mol. Sci..

[10] Yau J.W., Teoh H., Verma S. (2015). Endothelial cell control of thrombosis. BMC Cardiovasc. Disord..

[11] Kolluru G.K., Bir S.C., Kevil C.G., Calvert J.W. (2012). Endothelial Dysfunction and Diabetes: Effects on Angiogenesis, Vascular Remodeling, and Wound Healing. Int. J. Vasc. Med..

[12] Hanff T.C., Mohareb A.M., Giri J., Cohen J.B., Chirinos J.A. (2020). Thrombosis in COVID-19. Am. J. Hematol..

[13] Thurston G., Daly C. (2012). The Complex Role of Angiopoietin-2 in the Angiopoietin-Tie Signaling Pathway. Cold Spring Harb. Perspect. Med..

[14] Neuhauß A.K., Gutbier B., Friedemann T., Günther A., Doris Stoll D. Angiopoietins: Possible Biomarkers in Severe Pneumonia?|European Respiratory Society. https://erj.ersjournals.com/content/40/Suppl_56/P830.

[15] Akwii R.G., Sajib M.S., Zahra F.T., Mikelis C.M. (2019). Role of Angiopoietin-2 in Vascular Physiology and Pathophysiology. Cells.

[16] Volleman C., Ibelings R., Vlaar A.P.J., Brom C.E.v.D., van Agtmael M.A., Algera A.G., van Amstel R., Appelman B., van Baarle F.E.H.P., Bax D.J.C. (2023). Endothelial Permeability and the Angiopoietin/Tie2 System Following Mild and Severe COVID-19. Artery Res..

[17] Fang Y., Li C., Shao R., Yu H., Zhang Q., Zhao L. (2015). Prognostic significance of the angiopoietin-2/angiopoietin-1 and angiopoietin-1/Tie-2 ratios for early sepsis in an emergency department. Crit. Care.

[18] Flaumenhaft R., Enjyoji K., Schmaier A.A. (2020). Review Series Vasculopathy in COVID-19. Blood.

[19] Lázaro A., Zaranza M., Meneses G., Aragão N., Freire M., Guimarães Á.R., Beliero A., Dantas M., Forte L., Martins A. (2023). Predictors of mortality in critically ill patients with COVID-19 and diabetes. Braz. J. Med Biol. Res..

[20] Baaten C.C., Vondenhoff S., Noels H. (2023). Endothelial Cell Dysfunction and Increased Cardiovascular Risk in Patients with Chronic Kidney Disease. Circ. Res..

[21] Figuer A., Alique M., Valera G., Serroukh N., Ceprían N., de Sequera P., Morales E., Carracedo J., Ramírez R., Bodega G. (2023). New mechanisms involved in the development of cardiovascular disease in chronic kidney disease. Nefrología.

[22] Abou-Arab O., Bennis Y., Gauthier P., Boudot C., Bourdenet G., Gubler B., Beyls C., Dupont H., Kamel S., Mahjoub Y. (2021). Association between inflammation, angiopoietins, and disease severity in critically ill COVID-19 patients: A prospective study. Br. J. Anaesth..

[23] Vassiliou A.G., Vrettou C.S., Keskinidou C., Dimopoulou I., Kotanidou A., Orfanos S.E. (2023). Endotheliopathy in Acute COVID-19 and Long COVID. Int. J. Mol. Sci..

[24] Alay H., Laloglu E. (2021). The role of angiopoietin-2 and surfactant protein-D levels in SARS-CoV-2-related lung injury: A prospective, observational, cohort study. J. Med. Virol..

[25] Smadja D.M., Guerin C.L., Chocron R., Yatim N., Boussier J., Gendron N., Khider L., Hadjadj J., Goudot G., Debuc B. (2020). Angiopoietin-2as a marker of endothelial activation is a good predictor factor for intensive care unit admission of COVID-19 patients. Angiogenesis.

[26] Pine A.B., Meizlish M.L., Goshua G., Chang C.H., Zhang H., Bishai J., Bahel P., Patel A., Gbyli R., Kwan J.M. (2020). Circulating markers of angiogenesis and endotheliopathy in COVID-19. Pulm. Circ..

[27] Henry B.M., de Oliveira M.H.S., Cheruiyot I., Benoit J.L., Cooper D.S., Lippi G., Le Cras T.D., Benoit S.W. (2021). Circulating level of Angiopoietin-2 is associated with acute kidney injury in coronavirus disease 2019 (COVID-19). Angiogenesis.

[28] Villa E., Critelli R., Lasagni S., Melegari A., Curatolo A., Celsa C., Romagnoli D., Melegari G., Pivetti A., DiMarco L. (2021). Dynamic angiopoietin-2 assessment predicts survival and chronic course in hospitalized patients with COVID-19. Blood Adv..

[29] Kümpers P., Lukasz A., David S., Horn R., Hafer C., Faulhaber-Walter R., Fliser D., Haller H., Kielstein J.T. (2008). Excess circulating angiopoietin-2 is a strong predictor of mortality in critically ill medical patients. Crit. Care.

[30] Ong T., McClintock D.E., Kallet R.H.M., Ware L.B., Matthay M.A., Liu K.D.M. (2010). Ratio of angiopoietin-2 to angiopoietin-1 as a predictor of mortality in acute lung injury patients. Crit. Care Med..

[31] Gouzi F., Philippe A., Blervaque L., Günther S., Virsolvy A., Gruest M., Cazorla O., Rossi E., Smadja D.M. (2022). Plasma ratio of angiopoietin-2 to angiopoietin-1 is a biomarker of vascular impairment in chronic obstructive pulmonary disease patients. Angiogenesis.

[32] Seol C.H., Yong S.H., Shin J.H., Lee S.H., Leem A.Y., Park M.S., Kim Y.S., Chung K.S. (2020). The ratio of plasma angiopoietin-2 to angiopoietin-1 as a prognostic biomarker in patients with sepsis. Cytokine.

